# Development of Imaging System for Online Detection of Chicken Meat with Wooden Breast Condition

**DOI:** 10.3390/s22031036

**Published:** 2022-01-28

**Authors:** Seung-Chul Yoon, Brian C. Bowker, Hong Zhuang, Kurt C. Lawrence

**Affiliations:** U.S. National Poultry Research Center, USDA-ARS, Athens, GA 30605, USA; brian.bowker@usda.gov (B.C.B.); hong.zhuang@usda.gov (H.Z.); kurt.lawrence@usda.gov (K.C.L.)

**Keywords:** wooden breast, woody breast, detection, imaging system, chicken meat, poultry, myopathy

## Abstract

In recent years, the wooden breast condition has emerged as a major meat quality defect in the poultry industry worldwide. Broiler pectoralis major muscle with the wooden breast condition is characterized by hardness upon human palpation, which can lead to decrease in meat value or even reduced consumer acceptance. The current method of wooden breast detection involves a visual and/or tactile evaluation. In this paper, we present a sideview imaging system for online detection of chicken breast fillets affected by the wooden breast condition. The system can measure a physical deformation (bending) of an individual chicken-breast fillet through high-speed imaging at about 200 frames per second and custom image processing techniques. The developed image processing algorithm shows the over 95% classification performance in detecting wooden breast fillets.

## 1. Introduction

In the poultry industry, intensive genetic selection of broiler chickens, as well as advancements in nutrition and management over the years, have led to tremendous gains in bird growth rate, size, and muscularity. For broilers, the average market weight increased and the market age decreased by a factor of two in about 50 years [1,2]. Unfortunately, along with these advancements in live bird growth and performance have come an increasing occurrence of myopathies in these fast-growing modern broilers which have led to problems with the meat quality. The wooden breast condition (WBC) is a growth-related myopathy that occurs in the breast muscle (pectoralis major) of modern broilers that is of particular concern to the poultry industry due to its negative impact on breast meat quality and widespread occurrence [3,4,5]. Breast fillets exhibiting this myopathy have an uncharacteristically hardened or rigid palpatory feel and shape irregularities [3]. When consumed, meat with the WBC has been found to be notably less palatable than the normal meat [6] and consumer complaints about “rubbery” texture in cooked breast fillets have been increasingly linked to the WBC in raw products. Consumer complaints are forcing processors to redirect raw breast meat with varying degrees of the WBC into further processing and/or lower valued products, resulting in decreased yield and/or lost value.

In order to mitigate the problems presented by the WBC, there is a growing need to provide processors with methods and technologies for objectively and rapidly detecting and segregating fillets with WBC. Rapid and accurate detection of the WBC will allow processors to maintain the meat quality more uniformly through product sorting and to monitor their broiler production, handling, and slaughtering practices more accurately. The current industry practice to detect chicken meat with the WBC is a manual process using tactile characteristics (firmness, stiffness, etc.) and/or visual cues (a ridge-like bulge on caudal area, pale, clear, or slightly turbid viscous fluid cover, and/or petechial multifocal lesions) [3]. Manual inspection to detect the WBC is time-consuming, subjective, and prone to human errors. In trying to fully describe the WBC and develop better standards of separation, a considerable amount of research has been conducted on the meat texture characteristics of afflicted breast fillets using texture analyzers to measure the mechanical properties. Numerous studies have used compression force measurements and various types of shear force measurements in raw and cooked breast fillets to assess differences in meat hardness caused by the WBC [7,8,9,10,11]. Although instrumental evaluations of meat hardness have yielded some insight into the texture characteristics of the WBC, they are neither rapid nor nondestructive. Moreover, they do not provide objective ground-truth labels on meat categories for WBC. As a result, manual tactile and visual evaluation methods are still the most widely used gold standards for assessing the WBC in breast meat for both commercial processing plants and the poultry research community [8,9,11,12,13,14].

There is a large research gap in developing nondestructive and noncontact WBC detection technologies for commercial use [13]. An image analysis method for screening broiler carcasses in the shackle line was developed to predict the WBC in broiler carcasses with 84–91% accuracy for normal or WB classification such that 2D carcass shape features were extracted and analyzed [14,15]. The image analysis study [14] suggested that conformation changes in broiler carcasses were mainly related to a breast width increase as WB severity increased. Expressible fluid images were analyzed using deep learning to predict degrees of the WBC [16]. Image analysis was also used to measure intensity distributions and texture features in images of chicken breast fillets and combined with support vector machine for classification of normal or WB fillets with 91.8% accuracy, while near-infrared (NIR) spectroscopy showed higher performance with 97.5% accuracy [17]. A bioelectrical impedance analysis method was tested for its potential as a noninvasive biosensor for the WBC in breast fillets [18]. A study with NIR hyperspectral imaging used NIR spectral biomarkers associated with protein and water binding to predict the WBC [19,20]. A spectral domain optical coherence tomography (OCT) technique was used to measure differences in subsurface microstructures of normal and wooden breast fillets, where the epimysium thickness of the fillets with the WBC was about two times larger than the normal fillets [21]. Yoon et al. [21] also showed the potential of sensor fusion technology using OCT and hyperspectral imaging. Although these reported methodologies have shown some degree of potential for identifying breast fillets with the WBC, none are widely used throughout the poultry industry or research community.

In general, previous research sought to identify the WBC by measuring the biochemical, spectral, or optical properties of the broiler meat. Although the distinguishing characteristics of the WBC are largely described in terms of physical and mechanical properties (i.e., hardness and rigidity of breast meat), there have been no reports to date about online sensing technologies that can directly measure a physical or mechanical property of the breast meat to detect the WBC. Most studies on the mechanical properties of boneless breast fillets with the WBC have focused on the hardness characteristic, and the focus has not been in developing online sensing. To date, there have been no reports on the instrumental or tactile measurement of the rigidity or bending properties of boneless breast fillets with the WBC. Thus, the objective of this paper is to report the development of a machine vision system and image processing algorithms to measure and analyze the physical bending properties of boneless, skinless broiler breast fillets for the purpose of detecting the WBC.

## 2. Description of Imaging System

This section provides a description of the imaging system for wooden breast detection, developed in this study.

### 2.1. System Overview

The core idea of the imaging system was based on an observation that when a chicken breast fillet was moved and dropped over the end of a rolling pulley of a belt conveyor, the fillet would briefly be bent. If we can capture the shape deformations of a fillet near the discharging end of a conveyor with a high-speed camera, the captured pictures may contain the information about how a fillet is bent differently between normal and WBC fillets. Because belt conveyors are commonly used in the poultry processing plants, there are many places to observe this bending event. In particular, the shape of this bent fillet could be clearly viewed from the side of the conveyor system. Through this idea, a sideview imaging system was developed for capturing a series of pictures of a fillet while it was freely falling off the belt conveyor system at high speed. The captured individual images were processed and analyzed to measure the bending property of breast fillets with a developed image processing algorithm. This paper is the first formal scientific report of the patented technology [22].

### 2.2. System Description

**Figure 1:**
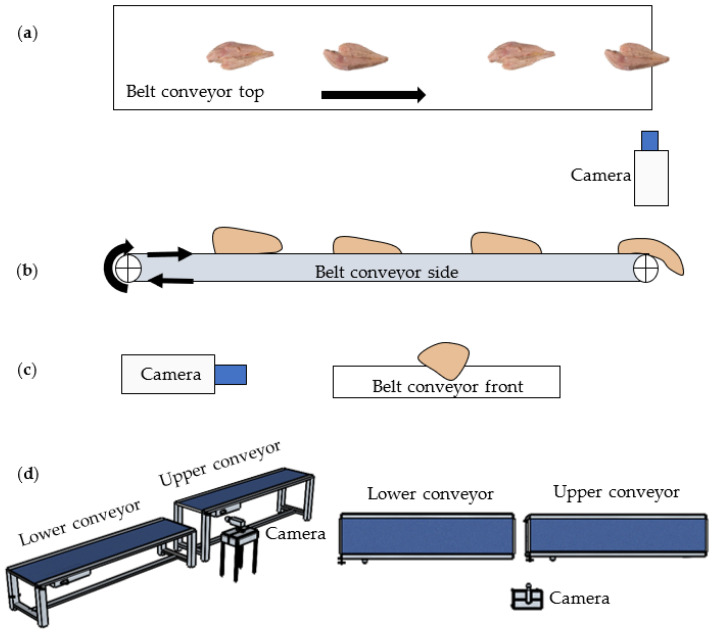
Schematics of the system setup viewed from (**a**) the top, (**b**) side, and (**c**) front; (**d**) schematics of the system with up- and downstream conveyors.

Figure 1a–c show the schematic diagrams of the sideview imaging system’s top, side, and frontal views for WBC detection, including a belt conveyor system and a machine vision system. Figure 1d also shows the system with the up- and downstream conveyors, where the fillets will travel from the upper conveyor and fall off onto the lower conveyor. Note that the lower conveyor was not implemented in the system reported in this paper.

**Figure 2:**
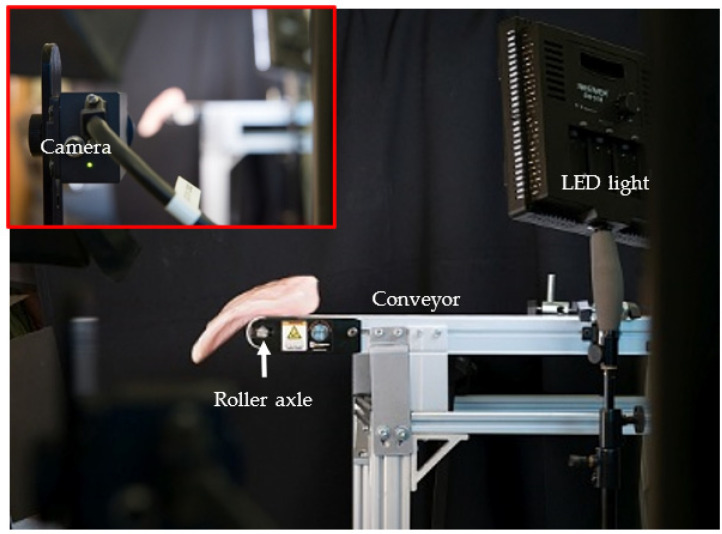
Picture of the developed imaging system with a fillet falling off the conveyor.

Figure 2 shows the pictures of the developed imaging system used in the paper. The imaging system consisted of a digital CMOS camera (Grasshopper USB 3.0, GS3-U3-23S6C-C, FLIR Integrated Imaging Solutions), two white LED panel lights (CN-576, Neewer), a black fabric backdrop (background), a computer (not shown), and custom software. A conveyor system (AS40, QC Industries) was equipped with a flat belt (18″ width × 48″ length), two adjustable guide rails, a brushless DC motor, a digital driver, and a variable speed controller.

Matlab (MathWorks) was initially used for data analysis and development of the detection algorithm. A custom C++ application program for real-time imaging was developed to acquire images of a moving fillet, apply the developed imaging processing algorithm, and make a final decision about the tested fillet in real time. A multithreaded application program was designed and implemented with the Microsoft Visual C++ 2015 to handle both real-time image capture and processing in parallel on a general-purpose computer (Microsoft Windows 10).

### 2.3. Imaging Condition

Three different conveyor line speeds at 10, 50, and 100 feet per minute (FPM) were used in this study. The camera frame rate was set to 200 frames per second (FPS) with exposure time of 3.5 ms. The 8-bit RGB color images were acquired with the global shutter mode. The color images were converted to grayscale images for further processing and analysis. The focal length of the lens was 6 mm. The pixel resolution of 960 (width) × 696 (height) pixels was set within the camera using its region of interest function. The camera’s viewpoint was adjusted to closely align with the roller axle axis. With a free run mode, the camera continuously acquired and transferred a sequence of images to a memory buffer (a circular better) implemented by the software application program, similar to our previous real-time spectral imaging system [23].

Individual fillets were manually placed in a single file with a spatial gap between them in the same surface and orientation of each fillet (a sample pose). From a preliminary study, we evaluated four different sample poses: (1) skin-side surface down and tail-end first, (2) skin-side surface down and head-end first, (3) bone-side surface down and tail-end first, and (4) bone-side surface down and head-end first. Note that the tail-end of a fillet is typically thinner than the head-end. From this preliminary study, we found that pose 1 (skin-side surface down and tail-end first) was the best sample pose. Thus, pose 1 was used throughout this study.

## 3. Description of Imaging Processing Algorithm

**Figure 3:**
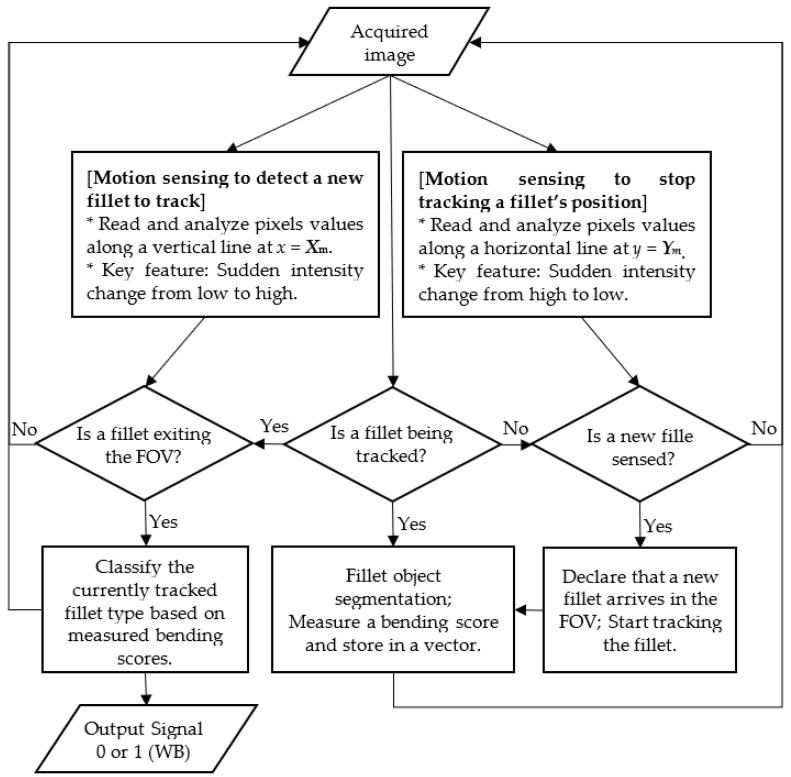
Flowchart of the developed image processing algorithm. FOV = field of view.

This section provides a description of the developed image processing system for woody breast detection. Figure 3 is a flowchart of the developed image processing algorithm that consists of image acquisition (previously described), motion sensing, filler object segmentation, measurement of fillet’s bending, and classification.

### 3.1. Motion Sensing

A sensor was necessary to trigger an event signal for beginning or ending of tracking and analyzing a filter’s shape deformation. Two optical sensors could be used to monitor the start and end events at two different locations and times. Instead, we developed an image processing algorithm, called a motion sensing algorithm, such that the algorithm emulated the motion sensing functionality of an optical sensor. A software motion sensor is more desirable in harsh washdown environments than hardware optical sensors due to its low cost, simplicity in system design, and no need for hardware maintenance. 

**Figure 4:**
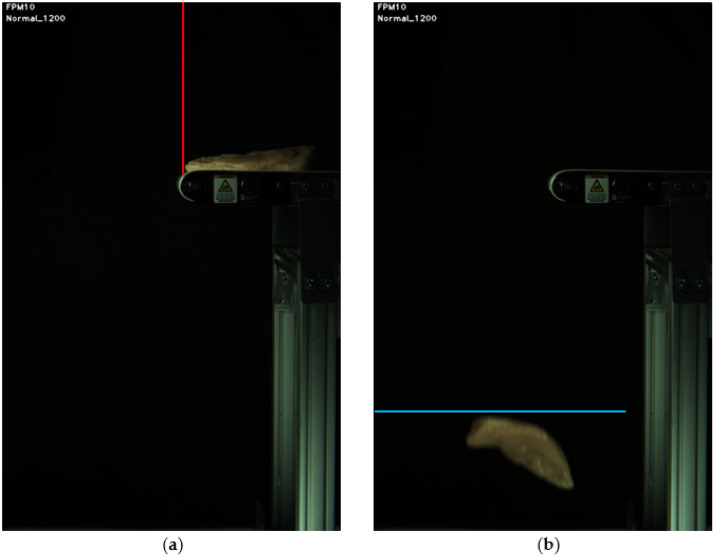
Software motion sensing trigger lines for detecting (**a**) new arrival (a vertical line) and (**b**) departure (a horizontal line) of a fillet for tracking and analysis.

For real-time motion sensing, pixel intensities along two lines (one column and one row) on an acquired image, called trigger lines, were analyzed. A software trigger signal (a Boolean flag) was generated when pixel intensities along the trigger lines exceeded predefined threshold values, respectively. The trigger lines were selected manually when setting up the imaging system, just once (see Figure 4). The first trigger line (a narrow vertical stripe of pixels with one-pixel width) was set to signal the arrival of a new sample. The second trigger line (Figure 4b) was a horizontal line (an image row) for detection of the fillet exiting the field of view (FOV).

### 3.2. Fillet Object Segmentation and Analysis

A sideview fillet object was obtained by an image segmentation algorithm, as follows: (1) global thresholding, (2) hole filling, (3) median filtering, (4) morphological opening, and (5) object size filtering. An object size filter was used to remove small, isolated objects. The image processing parameters for segmentation were obtained by trial and error using a calibration set of images that were collected during a system development phase. The developed image segmentation method generated a set of time-series sideview segmentation images from a fillet. 

The sideview shapes of a fillet were analyzed to characterize how the shape of the fillet changed over time. The motion sensor algorithm was set up to capture the first image of a fillet while the fillet was moving on the flat conveyor. This first image was used to extract the thickness and length information of the fillet. Two thickness definitions (maximum thickness and average thickness) were studied. Because the fillet’s thickness was estimated by an image analysis, fillet “thickness” and “height” are used interchangeably in this paper. The maximum thickness was the height of a bounding box enclosing a fillet segment. The average thickness was the average height of a fillet segment. The length was the width of the bounding box. The dynamic change (i.e., bending) captured in the fillet segments was extensively analyzed with two shape descriptors, described next in details.

### 3.3. Bending Shape Description by Bending Energy

**Figure 5:**
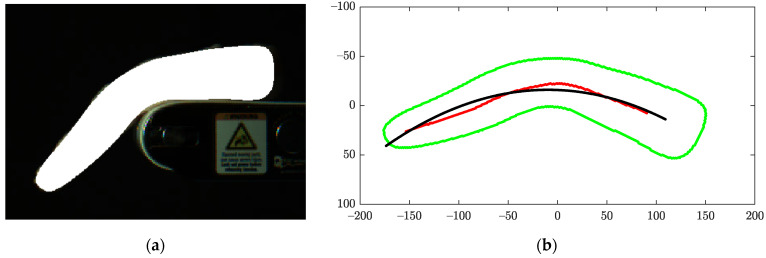
(**a**) Fillet object segmentation; (**b**) Raw (red) and fitted (black) skeletons on a rotated fillet shape contour.

A shape descriptor was developed to estimate a bending energy contained in a fillet shape contour. This shape measure quantified the energy stored in the shape of the contour. The bending energy of a fillet shape contour was defined as

(1) $$BE=\frac{P^{2}}{L}\sum_{k=1}^{L}C^{2}\left(k\right),$$

where *C* was the curvature of a curve passing through a bent fillet segment, *L* was the length of the curve whose value was closely related to the length of the fillet, and *P* was the perimeter of the segment contour. Note that the bending energy was normalized by *L* (the fillet length) and *P*^2^ (the squared shape perimeter of the fillet) to make it scale-invariant [24,25]. In theory, a neutral axis of a fillet, where neither extension nor compression occurred, was the best curve for Equation (1). However, because the neutral axes of real 3D physical fillets could not be accurately calculated from 2D images alone, a skeleton curve of a fillet segment was used as a more practical and approximate solution (Figure 5). The skeleton curve was obtained with the medial axis transformation and approximated with least squares polynomial fitting. The second-degree polynomial fitting was chosen after comparing the first- and up to fifth-degree polynomials. We hypothesized that a woody breast fillet would have a smaller bending energy when compared with a normal breast fillet, and the bending energy of a given fillet was maximized when its bending was maximized. The algorithm to predict the maximum BE of a fillet repeated the following steps.

(1) Given a fillet object segment (Figure 5a), the major axis of the fillet segment was extracted, and its angle was calculated. The centroid of the segment was also obtained.
(2) The fillet segment was rotated with the angle around the centroid such that the major axis was parallel to the *x*-axis (Figure 5b).
(3) The rotated fillet segment was skeletonized.
(4) The skeleton was regressed with a quadratic curve (second degree polynomial fitting).
(5) The *BE* was calculated with the regressed skeleton curve and the shape contour of the fillet segment.
(6) Repeated (1)–(5) until the motion sensor signaled the end of tracking.
(7) Once tracking was stopped, the maximum of all *BE*s calculated from the above steps became the maximum *BE* of the given fillet.

### 3.4. Bending Shape Description by Minimum Distance

**Figure 6:**
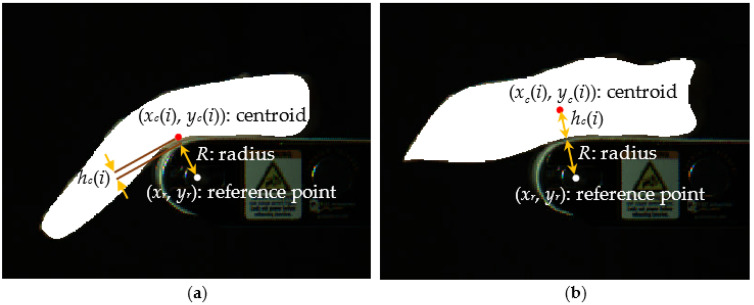
Parameters for distance measure *d_i_* = $\|\mathrm{centroid}\left(i\right)-{\mathrm{reference}}_{\mathrm{point}}{\|}_{2}$ shown on images of (**a**) normal and (**b**) severe woody breast fillets.

The next shape descriptor was based on a distance metric calculating the Euclidean distance between two special positions on an image. Suppose that a fillet was analyzed with *N* captured images and each image was segmented with the object-segmentation method. Then, the distance measure of *d_i_* on the *i*-th image was calculated with

(2) $$\begin{array}{l} d_{i}=\sqrt{{\left(x_{c}\left(i\right)-x_{r}\right)}^{2}+{\left(y_{c}\left(i\right)-y_{r}\right)}^{2}},\;\;\;i=0,\ldots ,\;N-1\\ =h_{c}\left(i\right)+R, \end{array}$$

where $x_{c}\left(i\right)$ and $y_{c}\left(i\right)$ were the coordinates of a centroid, $x_{r}$ and $y_{r}$ were the coordinates of a reference point at the center of the roller axle, *h_c_*(*i*) was a distance from the centroid to the conveyor belt surface point existing along the line connecting the centroid and reference point, and *R* was the radius of the roller axle as a constant (Figure 6). The reference point remained unchanged after it was manually determined during the system setup. The centroid was the center of mass of the fillet region from a binary segmentation image. The distance measure was based on the assumption that the value of *d_i_* for a fillet would become the minimum when its bending was maximized, and a normal breast fillet would have a smaller minimum *d_i_* than a woody breast fillet. 

**Figure 7:**
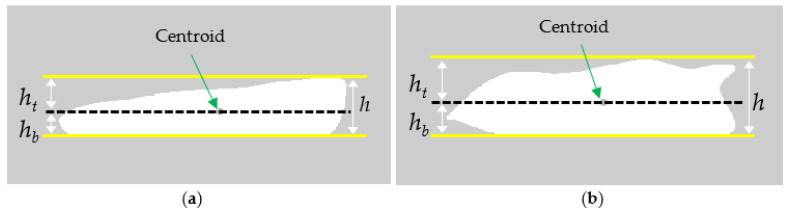
Height features of flat fillets for normalization of distance measure, shown on (**a**) normal fillet and (**b**) severe woody breast fillet.

Note that the distance measure of *d_i_* in Equation (2) was in the pixel unit and thus not scale invariant. The scale invariance was important to increase the robustness of the distance measurements when they could be potentially affected by different shapes and/or sizes of fillets. In general, the distance measures of thicker fillets were larger when compared with thinner fillets. If these thicker fillets truly had the woody breast condition, no problem occurred. However, a problem happened when those thick and thin fillets had very similar physical bending property but showed different distance measures (image properties) due to the different thicknesses and/or shapes. Hence, the shape and/or size of each fillet, such as thickness, needed to be factored in for normalization of the distance measurements. In this study, *d_i_* was normalized with a single scale factor of *H* proportionally according to thickness of a fillet, with a goal to develop a parsimonious model. With this goal in mind, the segmentation result from the first image for a fillet was used to obtain *H.* Note that the motion sensing algorithm parameters were set to ensure that the first image was captured while the fillet was still flat. From the first image, three different height features of h, $h_{t}$, and $h_{b}$ were obtained, where h was the maximum height, $h_{t}$ was the average height calculated from the fillet’s top bounding box down to the centroid, and $h_{b}$ was the average height measured from the bottom bounding box (a conveyor belt) up to the centroid (Figure 7). These height features were related to thickness of a fillet, depending on how thickness was defined. The height features were also related to each other with $h=h_{t}+h_{b}$. After performing a study to compare three different height features, the scale factor *H* of a fillet was defined as

(3) $$H=h_{t}+R\;\;\;\;\;\;\mathrm{at}\;i=0.$$

From Equations (2) and (3), the normalized distance measure ${\hat{d}}_{i}$ (unitless) was calculated for each image with

(4) $$\begin{array}{ll} {\hat{d}}_{i} & =\frac{d_{i}}{H}\;\;\;i=0,\ldots ,\;N-1\\ & =\frac{h_{c}\left(i\right)+R}{h_{t}+R}. \end{array}$$

The feature ${\hat{d}}_{i}$ was indirectly related to measurement of a ratio between $h_{t}$ and $h_{b}$ because $h_{c}\left(i\right)$ in the numerator *d_i_* was scaled from $h_{b}$ (see Equation (5) below). The denominator *H* was directly related to $h_{t}$ (Equation (3)). When we assumed a linear displacement of the centroid on the first image to the centroid on the *i*-th image, the following relationship held:

(5) $$h_{c}\left(i\right)=\alpha \cdot h_{b}+\beta \;∴h_{c}\left(i\right)\propto h_{b}$$

where α and β were unknown, for which α≤ 1 and $h_{c}\left(i\right)\leq h_{b}$. Estimation of α and β was beyond the scope of the study. Finally, the algorithm to obtain the minimum distance-based shape feature for a fillet is summarized below.

(1) From the segmentation result of the first image, *H* in Equation (3) was calculated.
(2) From the segmentation result of the *i*-th image, the centroid of the fillet region was obtained.
(3) The normalized distance measure ${\hat{d}}_{i}$ in Equation (4) was calculated, and steps 2 and 3 were repeated until tracking of the fillet was finished.
(4) Once the tracking was stopped, the minimum of all ${\hat{d}}_{i}$ was determined as the normalized minimum distance measure (NMDM) for the fillet.

## 4. Experimental Design and Analysis

### 4.1. Broiler Breast Fillet Samples

Approximately 200 boneless, skinless breast fillet samples (*Pectoralis major*) were obtained at 3 h postmortem from the deboning line of a commercial broiler processing plant. The samples were transported for ~1 h on ice to the U.S. National Poultry Research Center in Athens, Georgia, U.S.A. The samples were further trimmed of fat and connective tissue. Individual breast fillets were scored and categorized into normal (no WBC; WBC score 0), moderate WBC (WBC score 1), and severe WBC (WBC score 2) groups by a panel of trained experts using published criteria for tactile evaluation of fillet hardness and rigidity [9,11]. A total of 45 representative fillets (15 per each of three WBC scores) were selected for this study. Fillet weights were recorded, and the volume, maximum thickness, average thickness, and maximum length of each fillet were also measured by a structured light-based 3D scanner (Texas Instrument’s DLP^®^ LightCrafter 4500) built in-house. The physical properties obtained by the 3D scanner were computationally obtained through 3D point cloud processing in Matlab. Shear force was measured for each fillet using the blunt Meullenet-Owens razor shear (BMORS) method. For each fillet, seven shear measurements were measured on the ventral surface of the fillets using a texture analyzer (TA-XT-Plus, Texture Technologies Corp., Hamilton, MA, USA) with a 50 kg load cell [11,26]. Peak shear force (N) and total shear energy (N.mm) were analyzed. The 3D shape features and shear force for each fillet were measured after the data collection using the side-view imaging system.

### 4.2. Statistical Analysis and Performance Evaluation

For each fillet, seven physical features were measured: weight (g), volume (mm^3^), maximum thickness (mm), average thickness (mm), length (mm), peak shear force (N), and shear energy (N.mm). For each fillet, 11 image features were measured using the sideview imaging system: raw minimum distance measure (MDM), normalized minimum distance measure (NMDM), maximum bending energy (MBE), maximum height (MAXH), average height (AVGH), maximum length (MAXL), area (AREA), perimeter (PERIM), scale factor *H = h_t_ + R* (HTR), and alternative scale factor *H’ = h_b_ + R* (HBR).

The differences of each measured feature among fillet groups (normal, moderate WBC, and severe WBC) were analyzed with one-way analysis of variance (ANOVA) and post hoc tests. The Tukey test was used for the post hoc test. The statistical tests were performed with R (4.0.5) via RStudio (1.4). All studied features were one-dimensional data. Binary classification models in the 1D feature space were applied to divide the feature values into two groups (normal vs. WBC fillets). The evaluated classification models were the linear discriminant analysis (LDA), quadratic discriminant analysis (QDA), decision tree (DT), support vector machine (SVM), and k-nearest neighbors algorithms.

Note that three-class (normal, moderate WBC, and severe WBC) classifications were investigated but are not reported in this paper because the three-class classification results suggested that the severity of the WBC was difficult to predict with the developed methods and the paper length was limited. A 10-repeated five-fold cross-validation method was used to estimate the overall classification performance of each classification model and feature. The overall accuracy (OACC), balanced accuracy (BACC), F1 score (F1), and Matthews correlation coefficient (MCC) were the classification evaluation metrics. The OACC, BACC, and F1 had scores with a real number between 0 and 1, where 1 was the highest classification score and 0 was the lowest. The MCC had a score between −1 and +1, where +1 was the highest score (a perfect prediction) and −1 was the lowest (no prediction). The classification performance was evaluated with Matlab.

## 5. Results and Discussion

### 5.1. Imaging Results

**Figure 8:**
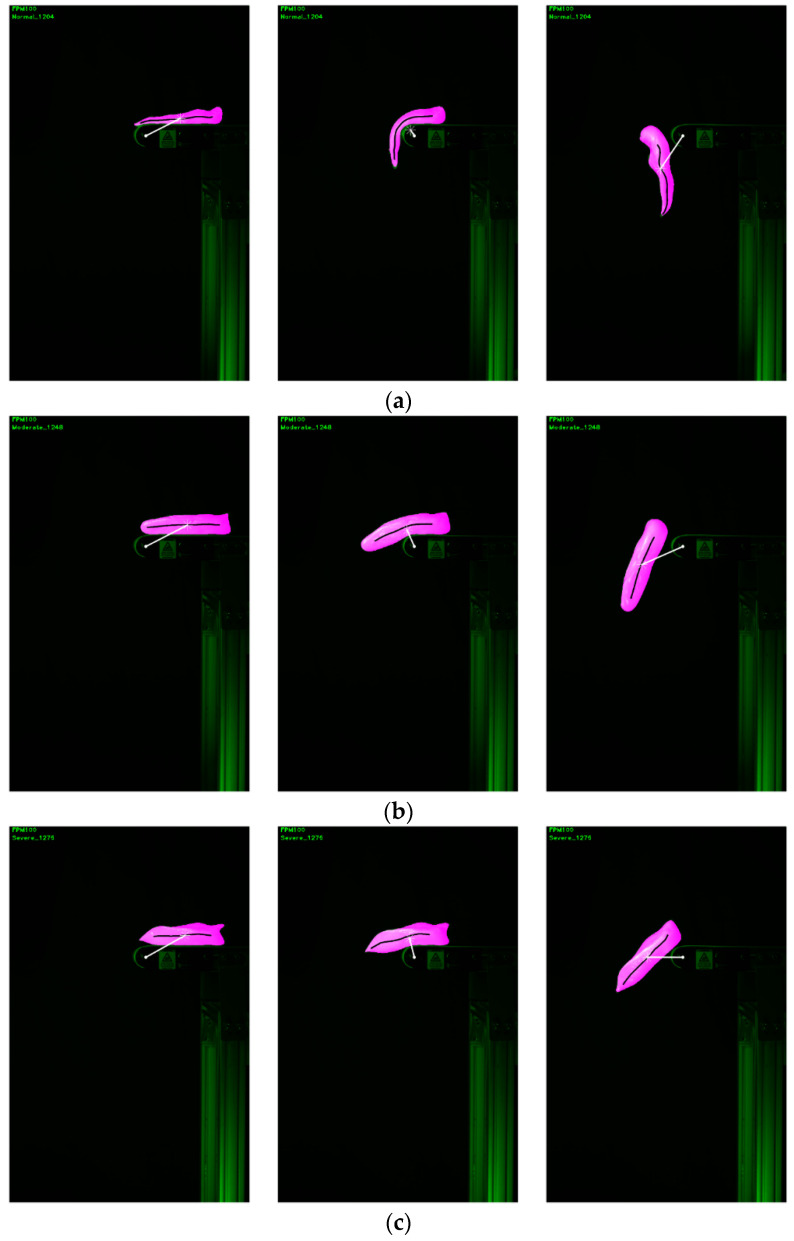
Example images on object segmentation (pink) of (**a**) normal, (**b**) moderate WB, and (**c**) severe WB fillets overlaid with the fillet centroids (white asterisk), center point of rotating axle (white dot), lines (white) connecting the centroid and the center point to calculate a distance, and fillet skeletons (black).

Figure 8 shows the images of representative fillets from arrival to the moment of maximal bending, and as they fell from the conveyor. The shape changes of typical normal, moderate WBC, and severe WBC fillets are clearly shown in segmented objects (colored pink in Figure 8) and the fillet segments in all images were successfully detected by the sideview image segmentation algorithm without error. In these example images, the segmented objects were overlaid to show the spatial contexts of each segment. The segmentation images showed that the shapes between normal and WBC (moderate and severe) fillets at their maximal bending moments were visually quite different while the difference between moderate and severe WBC fillets were not easy to recognize visually. The magnitude and direction of a measured distance metric are highlighted with a white line connecting a centroid and the reference point. 

**Figure 9:**
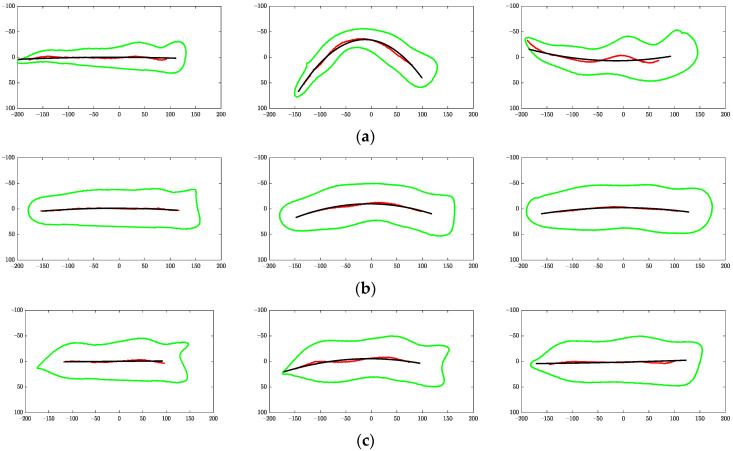
Objects to compute bending energy from regressed skeletons of (**a**) normal, (**b**) moderate WB, and (**c**) severe WB fillets. Raw skeletons are red, while regressed skeletons are black.

Representative skeletons used for the *BE* calculation are shown in Figure 9. The raw and fitted (regressed) skeletons in Figure 9 were based on the same example fillets used in Figure 8. 

### 5.2. Effects of Physical Features

Fillets in the WBC group (moderate and severe WBC fillets) showed greater average weight, volume, thickness, and shear force than normal fillets. Table 1 shows the average values of the physical features measured by three instruments (a scale, a 3D scanner, and a texture analyzer). The average values monotonically increased as the WBC severity increased (normal < moderate WBC < severe WBC). The means of the weight, volume, maximum thickness, peak shear force, and shear energy features were different between normal and WBC fillet groups (*p* < 0.05) but were not significantly different between moderate and severe WBC fillet groups. The post hoc test results of weights and BMORS values (peak shear force and shear energy) were consistent with previous findings [11,26,27] in that significant differences were observed only between normal and WBC fillets. The ANOVA and post hoc test results suggested that the average thickness could be good for group separability because all paired group means were significantly different from each other. On the other hand, the fillet length showed no significant differences between all pairs. The volume and maximum thickness features showed similar ANOVA and post hoc test results when compared with the weight and BMORS features. 

The Spearman correlation coefficients between each of the weight, volume, maximum thickness features and the WBC scores were *ρ* = 0.57, 0.63, and 0.61, respectively, with the *p*-value < 0.0001 (Table 1). The BMORS features (*ρ* = 0.66~0.67, *p* < 0.0001) were more strongly correlated with WBC scores than the weight, volume, and maximum thickness. Average thickness showed the strongest positive correlation with the WBC scores at *ρ* = 0.72 (*p* < 0.0001). From the ANOVA test results, it was not surprising that the correlation coefficient value of fillet length was quite low at 0.04 (*p* > 0.05). These results from 3D shape data implied that the effectiveness of average thickness could be attributed to the characteristic shape morphology of WBC fillets. The results also suggest the potential of 3D imaging as a tool to quantify the physical properties of the WBC condition. As a byproduct of side-view imaging, we could study some shape properties of the fillets, including average thickness, that we could previously only measure only with a 3D scanner.

### 5.3. Effect of Sideview Image-Based Shape Features

Table 2 shows the values of the shape features measured by the sideview imaging system. On average, the group means of all measured sideview image features except maximum bending energy (MBE) increased as the WBC severity increased (normal < moderate WBC < severe WBC). Note that the group means of MBE decreased as the WBC severity increased, as we expected based on the characteristic rigidity observed in WBC fillets. The ANOVA and post hoc test results of the raw minimum distance measure (MDM), maximum height (MAXH), average height (AVGH), and alternative scale factor (HBR) showed significant difference between all paired group means. These results were different from the instrumental measurement results of the physical features in that the average thickness was the only physical feature, where all paired group means were significantly different. The group means of the other image features (NMDM, MBE, AREA, and PERIM) except MAXL showed the same statistical trend as the volume, maximum thickness, and BMORS in that the means of normal and WBC fillet groups were significantly different but the group means of moderate and severe WBC fillets were not significantly different from each other. From the results obtained by both 3D imaging and side-view imaging, we could confirm that the fillet length was not a feature to consider when detecting the WBC fillets. 

The Spearman correlation coefficients between each of NMDM and MDM and the WBC scores were higher (*ρ* = 0.75~0.77, *p* < 0.0001) than the *ρ* values of any other instrumental measurements, including average thickness (*ρ* = 0.72) measured from the 3D data and shear force measurements (*ρ* = 0.66~0.67). The MBE feature had a negative correlation (*ρ* = −0.68) with the WBC scores. The rho value (*ρ* = 0.70) of the image-based average height (AVGH) was lower than, but close to, *ρ* = 0.72 of the average thickness (mm). This proximity of the rho value of the image feature (AVGH) to the rho value of the average thickness feature suggested that *ρ* = 0.70 could be a reference point for indirectly comparing correlation coefficient values between image-based and instrumental physical features. In other words, it implied that the distance measures, MDM (*ρ* = 0.77, *p* < 0.0001) and NDMD (*ρ* = 0.75, *p* < 0.0001), had the potentially best predictive ability among all proposed physical and image features. The statistical test results suggested that the raw distance measure (MDM) was the best feature in differentiating group means and correlation with the WBC scores, followed by the normalized distance measure (NMDM).

### 5.4. Analysis of Sideview Image Features Measuring Fillet Bending

**Figure 10:**
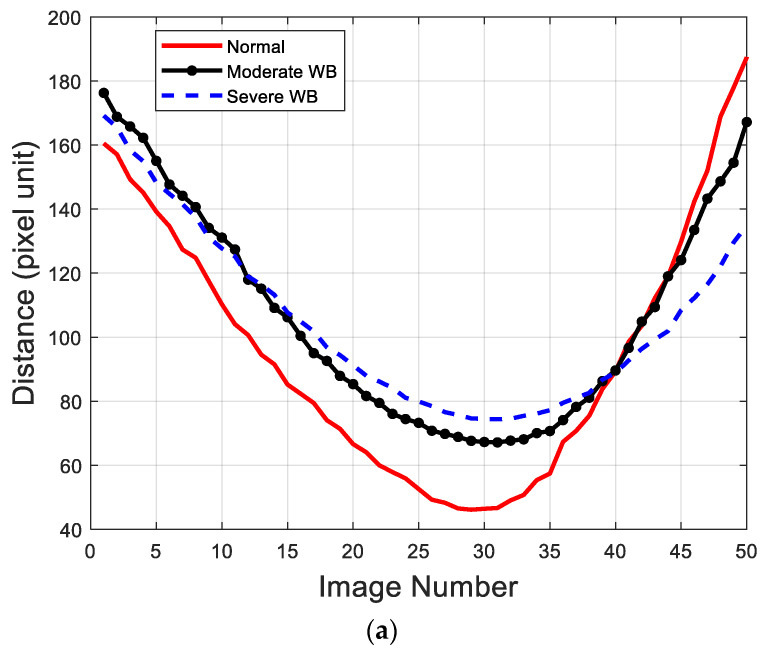

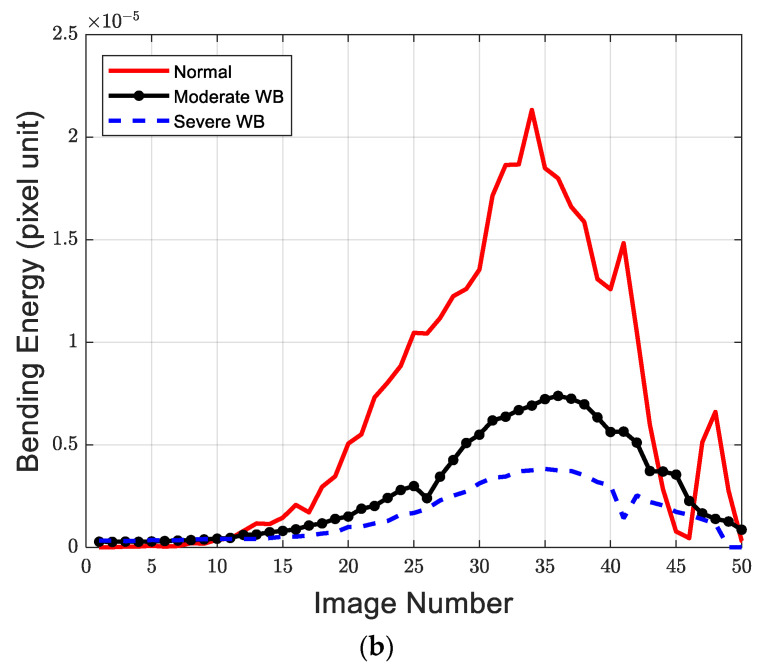
Raw data of measured (**a**) distance and (**b**) bending energy features in example normal, moderate WBC, and severe WBC fillets.

The values of the sideview image features (MDM, NMDM, and MBE) measuring the bending property were locally fluctuating yet globally convergent in that the developed iterative algorithms to find solutions were converged to global solutions. Figure 10 shows the curves of the raw distance and bending energy values measured with three example cases in Figure 8 and Figure 9. The curves in Figure 10 suggested that although maximal bending happened at about the 30th and 35th images for raw distance and bending energy measurements, respectively, the dynamic bending of fillets was well characterized by the sideview image features. As WB fillets experienced less bending than normal fillets, the WB fillets showed the smaller distance and larger bending energy values compared with normal fillets. Overall, the values of the distance measure were much less fluctuating locally when compared with those measured using bending energy. The curve analysis results suggested that the solutions to find the value of the minimum distance measure (MDM) were more stable when compared with bending energy. In the case of bending energy, the curves were generally more jagged than the curves of distance feature values but finding the value of the maximum bending energy (MBE) was also globally convergent. The local jaggedness of the bending energy feature was associated with a local change of a fillet shape during the dynamic bending process because the local change directly induced a change in detected skeletons. The results suggested that a more sophisticated image processing method to compute the skeletons invariant to small changes in shapes would be necessary to mitigate this issue. On the other hand, the solution to the distance-based search method was robust and insensitive to any sudden local deformations during the bending. Hence, these results suggested that the distance feature was a better metric to use when compared with the bending energy feature.

### 5.5. Performance of Classification Models in Predicting WBC

Preliminary results of the classification models for three-tier separation (normal, moderate WBC, and severe WBC) indicated that the studied features were less effective in distinguishing between moderate and severe WBC compared to the performance of binary classifiers for WBC and non-WBC. Thus, we report the results of the binary classification models for differentiating WBC from non-WBC fillets so that only the WBC fillets can be accurately detected and further sorted in practice.

Overall, the predictive performance of the sideview image-based distance measure and bending energy features was better when compared with the other measured features (Table 3, Table 4 and Table 5). The 10-repeated five-fold cross-validation (CV) test results showed that the QDA, SVM, and LDA yielded the higher scores of 0.831, 0.829, and 0.827, respectively, than the KNN (0.786) and DT (0.777) when all classification metric scores (OACC, BACC, F1, and MCC) were averaged over the 10 tested features and three line speeds (AVERAGE TOTAL in Table 3). Note that the scores for the different line speeds were obtained only with the sideview image features (NMDM, MDM, MBE, and AVGH). The repeated CV test results also showed that the SVM using the NMDM feature yielded the highest average score of 0.974, followed by the DT (0.962) and KNN (0.954) (Table 3).

The scores obtained by the SVM were further analyzed for each performance metric and feature category (Table 4). The NMDM feature yielded the highest mean (0.974) and individual scores in all performance metric categories with 0.98 (OACC), 0.973 (BACC), 0.985 (F1), and 0.956 (MCC), followed by the MDM (a mean score of 0.930) and MBE (a mean score of 0.882) when the weight and volume features yielded the lowest scores. The sideview image features (NMDM, MDM, and MBE) measuring the bending property yielded higher classification scores than the thickness, shear force, weight, and volume. The mean score (0.821) of the average thickness feature was higher than the mean score (0.812) of the maximum thickness feature. While the performance scores ranged between 0.98 (NMDM) and 0.9 (volume) for OACC, between 0.973 (NMDM) and 0.747 (volume) for BACC, between 0.985 (NMDM) and 0.858 (volume) for F1, and between 0.956 (NMDM) and 0.532 (volume), only the NMDM showed consistently very high scores over 0.95 in each performance metric category. The MDM also showed favorable performance scores (OACC = 0.947, BACC = 0.931, F1 = 0.961, and MCC= 0.882). 

The scores for the BE feature were lower than the NMDM and MDM but higher than the other features. The scores of the average thickness and shear energy features were similar to each other. The average thickness measured by 3D imaging yielded higher scores when compared with the AVGH (the average height of a fillet segment) and peak shear force. The scores of the maximum thickness feature were always lower than the score of the average thickness features. These results suggested that the original distance measure was probably affected by the shape and thickness of a fillet (e.g., a uniformly flat shape, a tapered shape, etc.) but the normalization scale factor *H* used in the NMDM feature increased the performance by reducing the effect of varying shape and thickness. The results also suggested that a more sophisticated shape feature describing the uneven surfaces and varying thicknesses of fillets would increase the detectability of the WBC fillets. These results were also aligned with the findings of the statistical significance tests in Section 5.2 and Section 5.3. We suggest that a sensor fusion of the sideview imaging with 3D imaging would solve this problem to increase the detection accuracy and potentially to discern the severity of the WBC. It is desirable to collect a dataset that will consist of more data reflecting the distribution of the real-world WBC sample population and assess the true performance of the technique with the dataset while testing the developed technology for a commercial use in the future.

Line speed affected the performance of the sideview image features (Table 5). Overall, the slower the line speed, the higher the performance score was. An average rate of change in performance scores at lower speeds (from 10 to 50 FPM) was much smaller when compared with the rate change of scores from 50 FPM to 100 FPM. On average, the performance scores at 10 FPM were reduced by 0.9% (OACC), 0.9% (BACC), 0.6% (F1), and 2% (MCC) when the speed increased to 50 FPM, while the scores were reduced by 5% (OACC), 9% (BACC), 4% (F1), and 13% (MCC) between 50 and 100 FPMs. The NMDM feature yielded the highest scores when compared with the scores of the MDM and MBE in all performance metric categories. At lower line speeds (10 and 50 FPMs), all performance scores of the NMDM were higher than or equal to 0.99. At 100 FPM, the NMDM showed still the highest scores of OACC (0.948), BACC (0.93), F1 (0.961), and 0.884 (MCC). The performance scores of the NMDM at 100 FPM were almost equal to the scores of the MDM at 50 FPM. The average reduction in performance scores of the NMDM was less than 0.5% when the line speed increased to the middle speed at 50 FPM from the slowest 10 FPM. The scores of the NMDM were decreased at much higher rates of 5% (OACC), 6% (BACC), 4% (F1), and 11% (MCC) on average when the line speed increased to 100 FPM from 50 FPM. The similar reduction in performance was observed from the tests carried out with the MDM and MBE as well. The MDM showed the least reductions in scores as the speed increased. The higher rate of change observed with the NMDM than the MDM was likely related to the small size of the dataset. The results suggested that a separate future study would be necessary to determine the maximum line speed that the sideview imaging system could handle.

### 5.6. Limitations and Future Work

It would be worthwhile to mention limitations of the current system presented in this paper and how to overcome it in practice. First, the travel requirement of fillets in a straight line may slow down the line speed. However, a dual-lane system using two sideview cameras and a matte black divider/blocker between lanes can increase the line throughput twice. Second, the current system is not equipped with a singulation system that can make all fillets travel in the same pose. One suggestion for a fully automated system is to recognize the current pose of a fillet through texture and shape analysis with an additional top-down machine vision camera and then change the pose mechanically as needed. For example, a paddle sorter or a diverter can be used to change the orientation of a fillet to move the tail-end first, and a flipper can be used to flip the surface of a fillet. Once the correct pose is set, individual fillets can be guided through side-guide rails such that the orientation change of a fillet is minimized. Third, a rejector needs to be installed to remove the WBC fillets, based on a decision signal from the imaging system. A paddle rejector or a drop-down rejector that is widely used in the food industry can be easily incorporated with the developed imaging system. Fourth, the developed technology cannot predict the severity of the wooden breast condition. This problem is a very challenging problem to solve due to the lack of the objective gold standard method and the huge variability of the wooden breast condition. One example of the variability is that the wooden breast condition may not be spatially uniform in fillets. The study finding implied that a sensor fusion of the sideview imaging and 3D imaging may provide a new insight into this problem. Fifth, a monochrome machine vision camera can replace the current color camera used in the study without loss of information.

## 6. Conclusions

The developed sideview imaging technology provided a new way of characterizing the woody breast condition in fresh broiler breast fillets. The study showed how to objectively measure the dynamic bending of boneless skinless breast fillets during poultry processing. The classification evaluation results showed that the sideview image features measuring the bending outperformed the conventional instrumental measurements including weight, volume, thickness, and shear force. The normalized distance measure was the preferred feature in differentiating the moderate and severe WBC fillets from the normal fillets. The study findings also suggest the potential of using a shape morphology (average thickness) in detecting the wooden breast fillets. The minimum distance measure feature produced overall accuracy of 95%, balanced accuracy of 93%, F1 score of 0.96, and Matthews correlation coefficient of 0.88 in detecting WB fillets moving at a high line speed of 100 feet per minute.

## 7. Patents

U.S. and international patents resulting from the work reported in this manuscript were granted on 24 March 2020 (US10596601B2 and WO/2018/213535). This manuscript is an expanded scientific paper of the patent application.

## Figures and Tables

**Table 1:** Instrumental measurements of physical features of fresh broiler breast fillets.

	WBC Category			Spearman Correlation Coefficient
Feature	Normal	Moderate WBC	Severe WBC	*ρ*
Weight (g)	388 ± 60 ^b^	489 ± 76 ^a^	499 ± 47 ^a^	0.57 ***
Volume (mm^3^)	329 ± 54 ^b^	426 ± 65 ^a^	442 ± 40 ^a^	0.63 ***
Maximum thickness (mm)	40 ± 5 ^b^	45 ± 3 ^a^	48 ± 3 ^a^	0.61 ***
Average thickness (mm)	25 ± 3 ^c^	32 ± 2 ^a^	33 ± 2 ^b^	0.72 ***
Length (mm)	185 ± 8 ^a^	188 ± 11 ^a^	189 ± 9 ^a^	0.04 ^ns^
Peak shear force (N)	11 ± 4 ^b^	24 ± 8 ^a^	27 ± 8 ^a^	0.66 ***
Shear energy (N.mm)	104 ± 28 ^b^	208 ± 69 ^a^	231 ± 70 ^a^	0.67 ***

^a–c^ Means within a row lacking a common superscript differ (*p* < 0.05). *** *p* ≤ 0.001, ^ns^ *p* > 0.05.

**Table 2:** Measurements of sideview image features of fresh broiler breast fillets.

	Category			Spearman Correlation Coefficient
Feature	Normal	Moderate WB	Severe WB	*ρ*
NMDM	0.41 ± 0.10 ^b^	0.63 ± 0.05 ^a^	0.66 ± 0.08 ^a^	0.75 ***
MDM (px)	41 ± 11 ^c^	63 ± 5 ^a^	67 ± 7 ^b^	0.77 ***
MBE	35 ± 20 ^b^	9 ± 5 ^a^	7 ± 7 ^a^	−0.68 ***
MAXH (px)	73 ± 8 ^c^	80 ± 7 ^a^	85 ± 7 ^b^	0.51 ***
AVGH (px)	49 ± 8 ^c^	62 ± 6 ^a^	65 ± 4 ^b^	0.70 ***
MAXL (px)	319 ± 12 ^a^	322 ± 18 ^a^	323 ± 15 ^a^	0.10 ^ns^
AREA (px)	16,498 ± 2628 ^b^	19,938 ± 2553 ^a^	21,113 ± 1784 ^a^	0.67 ***
PERIM (px)	745 ± 35 ^b^	783 ± 49 ^a^	787 ± 37 ^a^	0.40 ***
HTR (px)	99 ± 5 ^b^	100 ± 5 ^ab^	102 ± 6 ^a^	0.25 **
HBR (px)	84 ± 4 ^c^	89 ± 3 ^a^	92 ± 3 ^b^	0.68 ***

^a–c^ Means within a row lacking a common superscript differ (*p* < 0.05). *** *p* ≤ 0.001, ** *p* ≤ 0.01, ^ns^ *p* > 0.05.

**Table 3:** Performance scores of classification models: Results of 10 repeated five-fold cross-validations for each classification evaluation metric (OACC, BACC, F1, MCC) and at a line speed (10, 50, and 100 FPM only for MDM, NMDM, MBE, and AVGH) were averaged. The number in bold means the highest score.

	LDA	QDA	DT	SVM	KNN	MEAN
**Side-view imaging**						
NMDM	0.914	0.953	0.962	**0.974**	0.954	0.951
MDM	0.919	0.932	0.944	0.930	0.927	0.930
MBE	0.869	0.872	0.897	0.882	0.890	0.882
AVGH	0.819	0.810	0.752	0.812	0.780	0.794
**Avg. subtotal**	0.880	0.892	0.889	0.900	0.888	
**Instruments**						
Avg. thickness (mm)	0.839	0.855	0.798	0.821	0.836	0.830
Shear energy (N.mm)	0.832	0.832	0.786	0.813	0.738	0.800
Peak shear force (N)	0.813	0.804	0.741	0.807	0.740	0.781
Thickness (mm)	0.774	0.767	0.689	0.780	0.690	0.740
Volume (mm^3^)	0.761	0.759	0.599	0.734	0.642	0.699
Weight (g)	0.733	0.730	0.597	0.735	0.665	0.692
**Avg. subtotal**	0.792	0.791	0.702	0.782	0.719	
**Average Total**	0.827	0.831	0.777	0.829	0.786	0.814
**SD**	0.062	0.073	0.129	0.078	0.111	

**Table 4:** Performance of SVM model: Average of 10 repeated five-fold cross-validations. The results for MDM, NMDM, MBE, and AVGH were the averages over all line speeds (10, 50, and 100 FPM).

	Performance Score				
Feature	OACC	BACC	F1	MCC	MEAN
NMDM	0.980	0.973	0.985	0.956	0.974
MDM	0.947	0.931	0.961	0.882	0.930
MBE	0.908	0.893	0.932	0.794	0.882
Avg. thickness (mm)	0.864	0.830	0.902	0.689	0.821
Shear energy (N.mm)	0.851	0.843	0.886	0.673	0.813
AVGH	0.857	0.817	0.897	0.675	0.812
Peak shear force (N)	0.847	0.837	0.883	0.662	0.807
Max. thickness (mm)	0.833	0.792	0.880	0.614	0.780
Weight (g)	0.800	0.750	0.857	0.533	0.735
Volume (mm^3^)	0.800	0.747	0.858	0.532	0.734

**Table 5:** Performance scores of sideview image features over different line speeds (10, 50, and 100 FPM): Average of 10 repeated five-fold cross-validations.

Metric	Overall Accuracy			Balanced Accuracy			F1 Score			MCC		
FPM	10	50	100	10	50	100	10	50	100	10	50	100
MDM	0.960	0.948	0.932	0.957	0.938	0.900	0.970	0.961	0.951	0.910	0.884	0.851
NMDM	0.998	0.995	0.948	0.997	0.993	0.930	0.998	0.997	0.961	0.995	0.990	0.884
MBE	0.947	0.936	0.841	0.943	0.939	0.796	0.960	0.951	0.886	0.881	0.863	0.636
**MEAN**	0.968	0.960	0.907	0.966	0.957	0.875	0.976	0.970	0.933	0.929	0.912	0.790

## Data Availability

Not available.
